# Feminine Intimate Hygiene: A Review of Healthy and Unhealthy Habits in Women

**DOI:** 10.3390/medicina61071302

**Published:** 2025-07-19

**Authors:** Elizabeta Lohova-Matisa, Dace Rezeberga, Anna Miskova

**Affiliations:** 1 Gynaecology Clinic, Riga East Clinical University Hospital, Hipokrata Street 2, LV-1079 Riga, Latvia; dace.rezeberga@rsu.lv; 2 Riga Maternity Hospital, Miera Street 45, LV-1013 Riga, Latvia; anna.miskova@rsu.lv; 3 Department of Obstetrics and Gynaecology, Rīga Stradiņš University, Miera Street 45, LV-1013 Riga, Latvia

**Keywords:** hygiene, feminine hygiene, intimate hygiene

## Abstract

*Background and Objectives:* Intimate hygiene is essential in every woman’s cleaning routine and is strongly associated with women’s health. Unfortunately, there is minimal data available on correct intimate hygiene. *Materials and Methods*: This systematic review was conducted as per the Preferred Reporting Items for Systematic Reviews and Meta-Analyses (PRISMA) 2020 guidelines. Multiple electronic databases (PubMed, Embase, Web of Science) were searched. *Results:* We included 33 studies published between 2000 and 2022 that investigated washing habits, various products, underwear preferences, and pubic hair grooming habits among women. A total number of 21 (64%) articles discussed topics associated with washing habits, including douching, washing product usage, and the choice between bathing and showering. Out of 33 articles, 7 (21%) discuss underwear, clothing, and panty liner usage as a feminine, everyday intimate hygiene routine. A total number of 11 (33%) studies included pubic hair removal methods, reasons, and associated complications in their article. *Conclusions:* The majority of women have basic knowledge about correct intimate hygiene, including showering at least once a day and regularly changing their underwear. Douching remains one of the most popular intimate hygiene habits among women. Pubic hair shaving became the primary preferred grooming method.

## 1. Introduction

Intimate hygiene is crucial to women’s health, comfort, and self-esteem. It helps women feel fresh and clean daily. However, there is limited data on the proper practices and their effects on the genital microenvironment.

The vulva, which includes the mons pubis, labia majora and minor, clitoris, vestibule, vestibular glands, and bulb, is the first genital tract defence line against external physical, biological, or chemical factors [1]. The vulvar area can be divided into two parts by the type of epithelium that covers the genitalia. Keratinised, stratified squamous epithelium with sweat glands, sebaceous glands, and hair follicles can be found in the mons pubis, labia, clitoris, and perineum [1]. Despite keratinisation, the stratum corneum in this area is very thin and easily damaged, which can lead to easier penetration of microbes and other pathogens and substances into the deeper layers of the skin [2]. The second part includes vulvar vestibule mucosa, which is covered by non-keratinised stratified squamous epithelium and is even more vulnerable to external factors [1,3]. Although knowledge about the microbiome of the external genitalia is limited, there is no doubt about the significance of microbial ratios in supporting the health of the urogenital area. Vulvar microbiota is more complicated than initially thought and includes microorganism species found on the skin (e.g., *Staphylococcus epidermidis, Corynebacterium* spp.), of faecal origin (e.g., *Enterococcus faecalis*), of the vagina, and of urethral origin (e.g., *Lactobacillus* spp.) [4,5,6]. As a result of the anatomical, physiological, and microbiome variability of the vulva, its pH varies from 3.5 (vaginal pH) to 4.7 (skin pH) [7]. Moreover, various exogenous and endogenous factors can influence the changes in vulvar pH over a month and even within a day [8].

The vagina is a fibromuscular canal which connects the vulva and the cervix. Considering the close connection of the vagina with external genitalia and the influence of external trigger factors, more aggressive defence is necessary to protect the following organs—the uterus, fallopian tubes, ovaries, and abdominal cavities—from pathogens’ influence. The vaginal canal is covered by stratified squamous non-keratinised epithelium [1]. Several mechanisms, including the specific microbiome, pH, vaginal protective mucus, and normal discharge, are involved in protecting the vaginal mucosa and further organs from external trigger factors [9,10]. The vaginal microflora is dynamically changing, but *Lactobacillus* spp. remains the primary microorganism type in the vaginal microbiome [9]. During a woman’s reproductive age, oestrogen stimulates squamous epithelium proliferation and glycogen deposition in the epithelial cells of the vagina [11]. Later, glycogen is converted to glucose and used by Lactobacillus [11]. Glucose undergoes anaerobic metabolism, resulting in the formation of lactic acid [11]. This organic acid is a primary factor in creating and maintaining the acidic pH of the vagina, lowering it from a neutral pH of approximately 7 to a more acidic range between 3.8 and 4.4 [9,11]. Such a low pH is essential for vaginal health, as it serves as a protective barrier by inhibiting the growth and inflammatory potential of various pathogenic microorganisms [12,13,14]. Additionally, a protective layer of mucus is synthesised [10]. Discharge from the genitalia helps to remove desquamating, old epithelial cells and inactivated pathogens [10]. A woman’s hormonal levels decrease with age. The most significant decrease in hormonal levels is typically observed during menopause. Oestrogen level changes lead to a reduction in glycogen deposition in squamous epithelium, squamous epithelium proliferation, and mucus production in the vagina [15,16,17]. As a result, the number of Lactobacillus starts to decrease and is replaced by other microbiota (*Gardnerella vaginalis*, *Ureaplasma urealyticum*, *Candida albicans*, and *Prevotella* spp.); pH increases approximately to 7, reducing mucus production, which leads to vaginal dryness [15,16,17]. The primary defence mechanisms of the genital area become weaker, making it more vulnerable to pathogen influence.

Vulvovaginal hygiene is associated with a woman’s confidence, attractiveness, good health, and a satisfying sexual life. To support an adequate microbiome of the external and internal genital organs, it is necessary to perform qualitative, non-harmful, and healthy everyday intimate hygiene procedures. However, several factors influence every woman’s choice.

A hygiene routine is formed based on a woman’s personal preferences. However, factors such as religion, social life, and cultural norms significantly influence women’s preferences and habit formation [1]. Moreover, family members, such as mothers, grandmothers, and friends, teach women their hygiene routine habits [18,19]. The technology and social media development era has a significant influence on women’s choices of specific products [18,19]. Additionally, intimate hygiene norms are undergoing dynamic changes, and the number of intimate hygiene products is increasing rapidly. Nowadays, women are confronted with a broad spectrum of intimate hygiene products, and they should be able to choose the most suitable products for comfortable usage and to support their body health.

Unfortunately, some factors can give the wrong impression of a regular hygiene routine. Additionally, the effectiveness of some procedures and products can be highly questionable or negatively impact women’s hygiene habits and intimate health. An improper understanding of intimate hygiene can lead to inappropriate product usage or avoidance of intimate hygiene products, resulting in microbiome imbalance, pathogen proliferation, and abnormal pH levels. These changes increase the risk of different disease development, such as vaginal infection and microbiome disbalances (bacterial vaginosis, candida vaginitis) [18], increased risk of sexually transmitted diseases (*Neisseria gonorrhoea*, *Chlamydia trachomatis*, human immunodeficiency virus—HIV) [19], and even an increased risk of cervical cancer [20]. Unfortunately, genital microbiome changes can negatively influence future pregnancies and foetal development, leading to ectopic pregnancy, infertility, low birth weight, premature birth, and other complications [18,21,22].

This study aimed to review articles about women’s intimate hygiene products, habits, and their suitability for intimate feminine hygiene.

## 2. Materials and Methods

This systematic review was conducted based on the Systematic Reviews and Meta-Analyses (PRISMA) 2020 guidelines [23].

### 2.1. Eligibility Criteria

Specific inclusion and exclusion criteria were applied to select articles that aligned with the study’s purpose.

Inclusion criteria: (1) studies published between 2000 and 2022; (2) studies that included healthy women of reproductive age, pre-adolescents, adolescents, pre-menopausal and menopausal women; (3) studies investigating women’s intimate hygiene habits, different intimate hygiene product usage, and associated complications and complaints; (4) studies that included women with regular menstrual cycles.

Exclusion criteria: (1) systematic reviews; (2) meta-analyses; (3) studies published in non-English-language journals; (4) studies that included women with urinary tract infections; (5) studies that included pregnant women, breastfeeding women, and women with menstruation.

### 2.2. Search Strategy

Multiple electronic databases (PubMed, Embase, and Web of Science) were searched on August 8, 2023. Certain filters were applied to sift through the necessary articles, including a publication year filter, language filter, and an “article” type filter for clinical trials. We used the following keywords: “woman’s intimate hygiene”, “woman’s intimated hygiene habits”, “intimate area washing”, “intimate cleansing”, “intimate area shaving”, “underwear changing”, “underwear material”, “intimate liquid soap”, “vaginal douching”, “panty liners”.

### 2.3. Data Extraction and Analysis

Specific keywords were used to identify relevant articles (see “Search strategy”). Duplicates were removed. Title and abstract screening, full-text screening, and data extraction were performed by one independent reviewer (E.L.). An additional overview of data and a final decision were conducted by the study supervisor (A.M.).

For each study, the following data were collected: study design, setting, population, preferred intimate hygiene habits, factors influencing the choice of certain types of intimate hygiene, and complaints and side effects associated with the chosen hygiene type. Intimate hygiene habits were divided into washing, underwear, and hair removal in the intimate area. The “Washing habits” category included the following topics: douching, washing product usage, and the effects and frequency of bathing and showering. The “Underwear” category included underwear material, loose and tight clothes/underwear, underwear changing frequency, and panty liners. The “Pubic hair grooming” category included the following topics: pubic hair removal reasons, methods, and complications. Data are summarised as proportions.

Since the included studies were highly heterogeneous in terms of design, populations, outcomes, and measurement methods, a meta-analysis was not feasible. Therefore, we employed a narrative synthesis to summarise the findings, and formal sensitivity or heterogeneity analyses were not conducted.

## 3. Results

### 3.1. Study Selection and Study Characteristics

**Figure 1:**
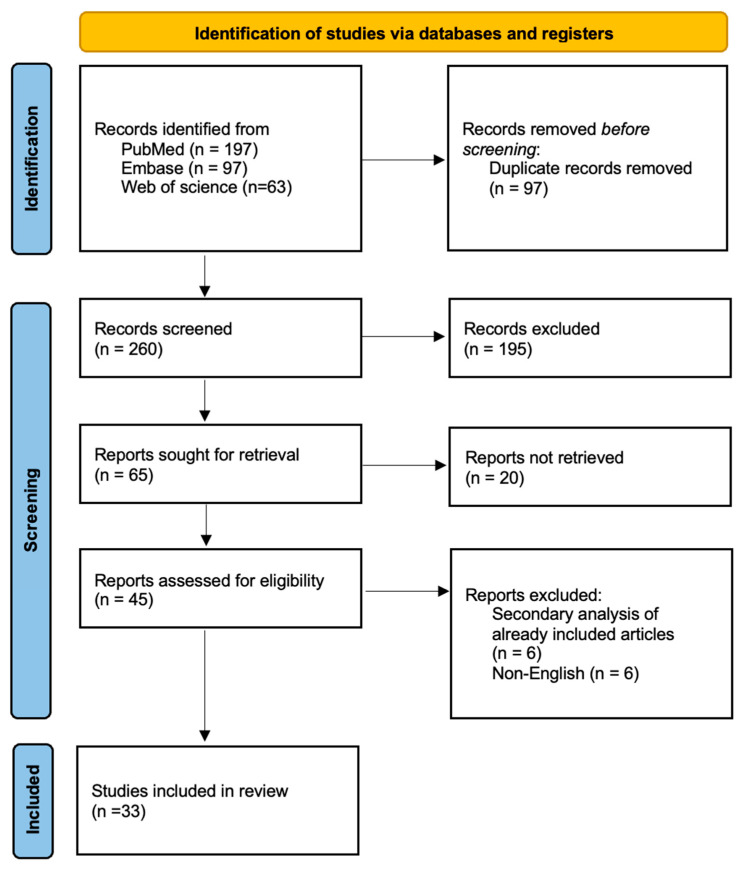
Preferred Reporting Items for Systematic Reviews and Meta-Analyses 2020 (PRISMA) flowchart of the study selection process.

The study selection process is summarised in a PRISMA flowchart (Figure 1). The PRISMA 2020 model was used to evaluate the study eligibility [23]. We screened 357 studies published between 2000 and 2022. Ninety-seven duplicate reports were excluded from the screening process. Studies that were excluded during title and abstract screening (*n* = 195) mainly involved pregnant women, breastfeeding women, women with menstruation, and women with urinary tract infections. Twenty articles were excluded due to a lack of full-text availability. During the full-text screening, six non-English articles and six secondary analyses of articles already included in the study, as identified by our search criteria, were excluded. Thirty-three studies with 23,991 participants were included [24,25,26,27,28,29,30,31,32,33,34,35,36,37,38,39,40,41,42,43,44,45,46,47,48,49,50,51,52,53,54,55,56] (Table 1).

### 3.2. Washing Habits

More than one-half of the studies (n = 21 (64%)) have discussed washing habits, including douching, washing product usage, and choosing between bathing and showering.

Of these 21 studies, in 15 studies (71%), douching, as an intimate hygiene habit, was discussed. Fourteen studies (67%) included women from the general population, and one study (5%) evaluated the douching habits of female gynaecologists. The reason for choosing douching as a part of intimate hygiene was observed in seven studies (33%). In all these studies, the main reason to douche was “for personal hygiene” or “to reduce infection possibility”. Meanwhile, an association of douching with different genital infections (bacterial vaginosis, *Candida albicans*, sexually transmitted diseases, and HIV) was discussed in 11 studies (52%). The increase in vaginal symptoms, such as itching, dyspareunia, and increased vaginal discharge, association with douching and its frequency was discussed in 4 out of 21 studies (19%). Only four studies (19%) evaluated the association between douching, education level, income level, and age.

In total, five (24%) studies included bathing and showering in discussion as a part of a feminine intimate hygiene routine. Out of these five studies, in four (19%) studies, it was observed that more than half of the women wash their genitalia once a day or often. Moreover, three (14%) studies found showering to be a preferred washing routine. Also, the association between bathing frequency and bacterial vaginosis or discomfort in the urogenital area was found in three (14%) articles.

The usage of different washing products and their influence on vulvar skin and vaginal microbiome were evaluated in 7 (33%) out of 21 studies. Four studies (19%) observed specific cleanser products that contained mainly natural components, such as salvia and chamomile, as well as non-irritating cleansers based on natural surfactants, lactic acid, and lactoserum. A positive influence on vaginal pH, vulvar humidity, and local symptoms, such as itching, burning, and skin changes, was detected. A comparison of vulvar cleaning with water and feminine gel/soap was made in the other three studies (14%). The last three studies did not disclose the names of the feminine gels/soaps used or their composition. An overview of the topics included in the selected articles is summarised in Table 2.

### 3.3. Underwear

Seven (21%) out of thirty-three articles evaluated underwear, clothing, and panty liner usage as a part of feminine intimate hygiene.

Only two (29%) articles discussed underwear material chosen as an essential factor in the feminine intimate hygiene routine. One study showed that approximately 80% of healthy women without symptoms or complaints choose to wear cotton underwear. The cotton underwear category includes pure cotton or a combination of synthetic materials. However, the second article found that women who douche or have bacterial vaginosis more often choose nylon underwear. Unfortunately, the regularity of underwear changing and bacterial vaginosis were included in only one (14%) study.

In total, two (29%) studies included loose and tight clothes and underwear as risk factors for vaginal symptoms and infection development. Tight underwear was preferred by women with bacterial vaginosis in one (14%) study. Interestingly, one of the studies showed that tight underwear was not associated with vulvodynia, but wearing tight jeans or pants at least four times per week increased the risk of vulvodynia development.

Different types of panty liners (breathable, non-breathable, regular, and string liners) and their influence on skin temperature, wetness, pH, and association with vaginal symptoms and discharge were compared in five (71%) studies. Out of these five studies, one study observed that 41% of panty liner users change them 4–6 times per day. Meanwhile, 83% of women who use panty liners complain about pruritus. Also, one of the studies has found a connection between douching and increased panty liner usage. No influence on the skin (lamina majora, perineum, interlabial fold) temperature, wetness, and pH was detected in non-panty liner users and breathable panty liner users in three (43%) studies. Moreover, significantly increased skin temperature, pH, and wetness were observed in non-breathable panty liner users in two (29%) studies. An overview of the topics included in the selected articles is summarised in Table 3.

### 3.4. Pubic Hair Grooming

A total number of 11 (33%) studies included pubic hair removal methods, reasons, and associated complications in their articles.

Pubic hair removal was mainly associated with the aesthetics of the intimate area, hygienic purposes, and self-confidence and was discussed in 5 (46%) out of 11 studies. Moreover, mainly friends, family members, and partners encouraged women to have their hair removed. Additionally, five (46%) studies observed that intimate area hair removal was significantly associated with younger age and an active sexual life.

Different pubic hair removal methods and preferences were discussed in nine (82%) studies. Despite the possibility of choosing from a wide range of removal methods such as shaving, waxing, depilatory cream, clippers/electric razors, tweezing, threading, and laser removal, shaving by using razors was chosen as a primary pubic hair removal method by approximately 60–90% of women (depends on the study) in seven (64%) studies. Only in two (18%) studies was waxing chosen as the primary method of hair removal. One of the last two studies observed hot pubic hair waxing as a leading choice among women’s gynaecologists.

In total, five (46%) studies included a section on post-pubic hair removal complications. In five (46%) studies, most women reported different clinical complications after hair removal, mainly after shaving. The most common complaints were about ingrown hairs (9–70%), severe itching (10–30%), and cuts (10–30%). Two case reports described the inflammatory process that occurs after the hair removal procedure. One of the studies reported a local inflammatory process, while the second study reported inflammation after waxing, accompanied by systemic signs. An overview of the topics included in the selected articles is summarised in Table 4.

### 3.5. Bias Analysis

Across all topic subgroups in this review, the number of included studies was fewer than 10. Therefore, we were unable to assess any reporting bias using funnel plots or statistical tests formally, and the level of concern remains unclear. However, we acknowledge the potential for reporting biases.

## 4. Discussion

Daily intimate hygiene is a specific ritual in every woman’s life that helps increase self-esteem, self-confidence, and feelings of attractiveness. If performed correctly, it is an integral part of supporting a woman’s health. Unfortunately, a lack of knowledge, old-fashioned habits, and a fear of asking questions about correct and healthy intimate hygiene can form an incorrect image of a regular daily hygiene routine and its influence on a woman’s body.

### 4.1. Washing Habits

The right approach to the daily washing routine significantly affects a woman’s well-being and confidence. The importance of washing habits in women’s daily lives is justified because 65% of studies have evaluated everyday washing routines, their benefits and harm, and possible complications [24,25,26,27,28,29,30,31,32,33,34,35,36,37,38,39,40,41,42,43,44].

After the studies’ data assessment, douching was shown as a part of a regular washing routine among approximately 30–70% of women [29,30,31,32,36,37,38]. Moreover, Ruiz et al. (2019) observed douching as a regular washing routine among 21% of Brazilian gynaecologists [25]. In relevant studies, a reason to include douching in a hygiene routine was explained as “to feel clean”, “to get rid of vaginal infection”, “to prevent vaginal infection development”, “to get rid of fishy smell”, and “to decrease itching and burning” [30,31,37,39]. As a result of douching’s popularity and the listed reasons to choose it as a part of a cleansing routine, possible misinformation about the normal vaginal microbiome and douching’s influence on it, as well as old-fashioned information passed to the new generation, caused the formation of such habits. McKee et al. in 2009 observed encouragement of women to start douching in 35.3% of cases by mothers and 50% by friends [37]. Moreover, Yildirim et al., 2020, detected a tendency to increase the rate of douching with a woman’s age [39]. Furthermore, the above-mentioned reasons to douche show a lack of understanding about the harm that douching can cause to the body. Relevant studies have shown an association between douching frequency and vaginal microbiome changes [32,36]. Microenvironmental changes in the vaginal area can lead to the development of candida vaginitis [25,27]. Moreover, douching increases sexually transmitted infections such as *Neisseria gonorrhoea* and *Chlamydia trachomatis* [26,32,34]. However, bacterial vaginosis is the most common complication of frequent douching [26,28,30,31,34]. Ness et al. (2001) showed that bacterial vaginosis was found in 50.8% of douching women compared with 28.2% of non-douching women [28]. However, the risk of bacterial infection is high, and douching increases the likelihood of transmitting viral infections. McClelland et al. in 2006 observed an increased risk of acquiring HIV-1 among women who douche compared with non-douching women [35]. Misconceptions about douching remain a severe problem, as it hurts women’s health. It is necessary to educate women about the influence of douching on the body and health.

Improvements in daily washing habits by excluding douching can significantly influence a woman’s well-being. However, giving up douching does not mean having appropriate, non-harmful washing habits because even elementary actions such as the inappropriate frequency of washing or unsuitable product usage can meaningfully influence health.

Shower and bath remain the two primary daily washing methods among women. Relevant studies have shown that showering has been chosen by 80–90% of women as a daily cleansing method [30,44]. Mościcka et al. (2020) observed changes in the frequency of showering during the SARS-CoV-2 pandemic, showing an increase in showering twice a day instead of once from 22.9% to 30% [44]. Moreover, a decrease in the number of women who choose bathing as the daily cleansing method was observed [44]. The border between the normal microbiome of the genitalia and the pathological one is very fragile. It is challenged every day by women’s washing routine preferences, frequency, and different intimate hygiene product usage. Holzman et al. (2001) found an association between bathing and showering frequency and bacterial vaginosis [30]. Bacterial vaginosis was more often observed in women who bathed frequently, at least four times per week [30]. Interestingly, a decreased frequency of bacterial vaginosis was detected among women who showered at least four times per week [30]. Also, Klann et al. (2019) found no association between washing method, water temperature, and vulvodynia [24]. Meanwhile, women with vulvodynia were mostly observed to wash their genitalia with only water [24]. Vulvar skin is susceptible and can be easily irritated by inappropriate cleansing products, leading to vaginal symptoms and the development of infections. Crann et al. (2018) found an increase in bacterial vaginosis and yeast infection development among the users of gel sanitisers, feminine wipes, feminine gel, and washes [26]. Relevant studies have shown that more suitable cleansers have a positive influence on the vaginal microbiome [27,40,41,43]. Vincenzo De et al. (2015) observed the antibacterial, antimycotic, anti-inflammatory, and antioxidant effects of daily intimate cleansers containing salvia and chamomile extracts [27]. Murina et al. (2020) tested two intimate products, one of which was based on natural extracts. Meanwhile, the other was a standard lactic acid product [40]. Both products showed adequate hydration and pH stability of vulvar skin [40]. However, cleansers can not only help support the normal microbiome of the genitalia but also may help reduce specific symptoms and lower the risk of infection development. Lactic acid plus lactoserum liquid soap could prevent bacterial vaginosis recurrence in women after oral metronidazole [41]. Moreover, non-irritating cleansers based on natural surfactants decrease burning and effectively treat vulvar dermatosis [43]. Intimate wash products with natural extracts and lactic acid support the microbiome of the intimate area, but more research is needed to confirm their effectiveness. Both cleaning methods and products can affect the microbiome and vaginal health.

### 4.2. Underwear

Women very often underestimate the influence of underwear type, material, and changing frequency on genital wellness. Unfortunately, a limited number of studies include underwear specifics as possible factors in genital microbiome changes, perineal skin irritation, and the development of different infections. After data analysis, only 21% of studies evaluated the specifics of underwear, thigh clothing wearing, and the association with panty liners and perineal area irritation and vaginal symptoms [24,25,33,42,45,46,57].

A variety of natural and synthetic materials are used in the manufacturing of underwear. The most popular materials are cotton and bamboo, as well as nylon and polyester, which are synthetic. Choosing the right underwear can be challenging for many women, as the material and design are crucial factors in making a decision. However, despite several factors influencing women, Ruiz et al. (2019) found that 85% of women preferred cotton as one of their primary underwear materials [25]. Natural materials, such as cotton and bamboo, are among the best choices for panties due to their superior water vapour permeability, moisture absorption, and deodorising properties compared to synthetic materials [57]. All these and more qualities are important for supporting the normal microflora of the perineal and vaginal epithelium. Although underwear material has not been directly associated with changes in the epithelial microbiome, synthetic underwear is a risk factor that increases the likelihood of changes in the epithelium. Klebanoff et al. (2010) studied hygienic behaviour associated with bacterial vaginosis [33]. The study showed an increase in bacterial vaginosis among women who douched and chose nylon underwear compared to women who douched and wore cotton or other material underwear [33]. Unfortunately, a limited number of studies have evaluated underwear material influence on the normal perineal and vaginal microbiome. However, it is possible to conclude that the material of underwear alone does not have a significant impact on the microbiome of the genitalia, and only a combination of multiple risk factors can significantly increase the likelihood of changes in the perineum and vaginal microflora.

Underwear material is not the only parameter that influences a woman’s perineal area. Wearing too-tight underwear and pants can alter the bacterial balance and cause physical damage to the skin. Klann et al. (2019) observed an increase in vulvodynia symptoms among women who wear thigh jeans or pants at least four times per week [24]. Bahram et al. (2009) studied 500 non-pregnant Iranian women’s hygiene habits associated with BV [42]. Interestingly, 60% of women with a BV diagnosis preferred to wear tight underwear. Meanwhile, 72–82% of healthy women chose loose underwear [42]. In conclusion, loose underwear and pants are more suitable for supporting a woman’s health; meanwhile, tight, fancy underwear should be avoided and is acceptable only for specific occasions.

Panty liners are advertised as easily usable, comfortable, healthy, everyday hygiene products widely used worldwide. However, most women have limited knowledge about the appropriate usage and different types of panty liners. A wide range of panty liners is produced and sold daily, each with distinct characteristics. Schafer et al. (2002) demonstrated that hygiene products impermeable to water vapour increase skin water loss and humidity, resulting in mechanical and chemical skin irritation [45]. Furthermore, Runeman et al. (2003) compared the influence of breathable and non-breathable panty liners on the perineal skin and mucosa [46]. The control group (no panty liners) and breathable panty liner group showed no difference in skin temperature, water loss, electrolyte balance changes, and pH [46]. Moreover, the non-breathable panty liner group showed increased skin temperature, skin surface moisture, and pH of the vulvar skin, thereby significantly changing the vulvar microbiome [46]. Although there was no difference between the no panty-liner group and the breathable panty-liner group, some studies have found that women who wear panty-liners daily and regularly change them have specific complaints. Ruiz et al. (2019) showed that 83% of women who wear panty liners every day and change them 4–5 times per day have complained about pruritus [25]. According to the mentioned study, a broad spectrum of different panty liner types is found in shops. Moreover, most women are unaware of the kind of panty liners (breathable or non-breathable) they use daily. However, choosing panty liners as everyday intimate hygiene products can be associated with already existing microbiome changes, mainly increased vaginal discharge. Klebanoff et al. (2010) found that panty liners are more prevalent among women who douche [33], and, as mentioned earlier, douching is significantly associated with vaginal microbiome changes, an increase in vaginal discharge amount, and vaginal symptom development. It is not recommended to wear panty liners every day unless necessary.

### 4.3. Intimate Area Hair Removal Methods

Pubic hair grooming is a common practice among women. Over time, pubic hair grooming tendencies have changed, and they are now significantly different from what they were 20–30 years ago. After the data assessment, 33% of the studies included topics related to pubic hair removal methods, reasons, and associated complications in their articles [24,25,48,49,50,51,52,53,54,55,56].

Most women prefer to remove their pubic hair partially or fully. Relevant studies found an association between pubic hair removal, younger age, and sexual activity [49,50,51,52,55,56]. Herbenick et al. (2010) surveyed 2451 American women about their pubic hair removal habits [55]. As a result, pubic hair removal was preferred by 60–70% of women in the 18–40 age group; meanwhile, less than 50% of women older than 50 chose to remove their pubic hair [55]. DeMaria et al. (2014) showed that 59% of women removing their pubic hair were in the 21–30 age group [49]. Interestingly, the majority of women continued to remove their pubic hair despite some complications, explaining it by “increased self-confidence”, “feeling more attractive”, “hygienic”, “more comfortable”, “visually pleasing or sexually arousing partner”, and “reduced irritation during sexual intercourse” [24,25,48,51,52,55]. Summarising the reasons to remove pubic hair, it can be concluded that women’s self-confidence and sexual satisfaction lead to the choice of pubic hair grooming.

Numerous hair removal methods are available, such as shaving, clipping, epilation, depilatory creams, laser hair removal, electrolysis, and sugaring. Women prefer quick, pain-free, easy, and cheap methods to groom or remove pubic hair. Shaving was the most preferred method of pubic hair removal among women, but its popularity varied from study to study. Relevant studies have found that 75–99% of women prefer using razors [49,51,52,56], and approximately 80–90% perform this procedure at home [48,51,52]. Rouzi et al. (2018) evaluated hair removal practices among Saudi women and found that 33.5% of women preferred only razors to remove pubic hair [48]. However, 41.8% chose to use multiple methods simultaneously to achieve the desired result [48]. Attractive preferences for pubic hair grooming methods among Brazilian gynaecologists differ from those of the population. Ruiz et al. (2019) observed that gynaecologists more often choose hot wax (40.9%) compared with shaving (29.5%) [25].

Several different factors influence the decision of pubic hair grooming and removal type. Many studies have observed family members, especially mothers, friends, and social media, encouraging women to start styling and removing their pubic hair at a younger age [48,51,52]. Unfortunately, incorrect information about pubic hair grooming from misleading and unconvincing sources can lead to different mild to severe complications. Approximately 60–70% of women reported various clinical complications after hair removal, primarily after shaving [48,49,51,53,54]. The most common complaints were ingrown hairs (9–70%), severe itching (10–30%), and cuts (10–30%). Rouzi et al. (2018) evaluated the practices and complications of pubic hair removal in Saudi women [48]. Although 75.5% of women complained about cuts, ingrown hair, severe itching, and other complications (including rash, allergies, bruises, and abrasions), 17.9% of women sought medical attention in the hospital and required treatment for complications associated with their pubic hair removal [48]. Not only does the pubic hair removal method define the type of possible complications, but also women’s comorbidities, which are associated with slower wound healing, increased possibility of infection development, and other more severe complications. Dendel et al. in 2007 presented a case report about a 20-year-old woman with poorly controlled type 1 diabetes mellitus [54]. The woman has developed life-threatening *Staphylococcus pyogenes* and *Herpes simplex* infections after “Brazilian” waxing [54].

### 4.4. Guidelines on Feminine Hygiene

Intimate feminine hygiene topics are often under-represented in medical studies, significantly limiting information availability. The last two guidelines that included feminine hygiene were published in 2011. The Royal College of Obstetricians and Gynecologists (RCOG) conducted an extensive literature search and analysis to prepare evidence-based guidelines on managing vulvar skin disorders, where feminine hygiene was part of the treatment and symptom relief methods [58]. The Middle East and Central Asia (MECA) region developed the second evidence-based guideline, covering topics related to female genital hygiene [59]. Both guidelines recommend using a specific gel for washing the intimate area. It is better to avoid regular soap bars, shower gels, bubble baths, scrubs, or just plain water, as they can irritate or dry out vulvar skin. Additionally, wearing loose-fitting underwear and clothing made from cotton or other breathable materials is recommended [58,59].

The RCOG guidelines also suggest avoiding coloured underwear, as dark-coloured clothes (black, navy) can irritate the skin or cause an allergic reaction [58]. It is also better to avoid coloured toilet paper [58]. The MECA guidelines additionally suggest avoiding douching and choosing a safe method of pubic hair removal to decrease the possibility of sensitivity and scarring [59].

### 4.5. Strengths and Limitations

The strengths of this systematic review include our comprehensive search strategy, the use of multiple electronic databases, and the selection of the most recent articles and case reports. Our study has several limitations. As several feminine hygiene topics are not widely studied or actively included in medical studies, a limited number of articles, or even no articles, were found on specific issues. A limited number of studies explored topics such as washing frequency and different product usage, the influence of underwear material on the skin, and the frequency of underwear changes. This may contribute to additional heterogeneity in the results. For instance, the absence of information about underwear changing frequency and underwear material prevents the evaluation of the association between underwear habits, vaginal symptoms, and the development of perineal skin irritation with certainty. Another limitation is that we filtered our studies based on time and type. Although the time frame expansion and inclusion of older studies would provide more data and possibly cover topics with limited information, the data quality would be poorer. It would increase the possibility of misconceptions about intimate hygiene, as feminine hygiene habits change rapidly.

## 5. Conclusions

The majority of women who follow specific healthy intimate hygiene rules, such as showering regularly, choosing cotton underwear, wearing loose underwear and pants, avoiding douching, and not using panty liners every day, rarely showed complaints about increased or smelly vaginal discharge, vaginal symptoms, and vulvodynia. Additional studies are necessary to evaluate the preferred washing product usage during showers, the frequency of underwear changes, and preferences for panty liner types.

Douching remains one of the most common intimate hygiene practices among women and is significantly linked to changes in the vaginal microbiome and the development of infections. Young women, their family members, and friends should be more carefully educated about the possible harm and complications of douching.

Younger women often choose to shave their pubic hair as a primary grooming method, but most are unaware of the potential complications associated with pubic hair shaving. Additional educational information should be disseminated to younger women about selecting the most suitable pubic hair removal method and possible complications associated with specific procedures.

## Figures and Tables

**Table 1:** Summary of included articles.

Article (Author, Year)	Study Design	Country	Participant Number	Population	Topic	Results	Quality
Bahamondes et al. (2011) [41]	Prospective cohort study	Brazil	123	Women with bacterial diagnoses	Lactic soap post-metronidazole to prevent BV recurrence	The use of a lactic acid plus lactoserum liquid soap for external intimate hygiene may be an effective option for preventing BV recurrence after oral metronidazole treatment. Significant improvements in quality of life were noted, indicating potential benefits beyond infection prevention.	O
Bahram et al. (2009) [42]	Descriptive-analytic cross-sectional study	Iran	500	Non-pregnant married women	BV and hygiene	BV prevalence among non-pregnant Iranian women (16.2%) was substantial. Statistical analysis showed a significant correlation between BV menstrual status and individual vaginal hygiene (*p* < 0.01 and *p* < 0.001, respectively). In contrast, no significant correlation was observed between BV and coital hygiene. Genital and menstrual hygiene practices appeared to influence BV risk significantly. Education programs on hygienic behaviours may help reduce BV prevalence in similar contexts.	O
Butler et al. (2015) [50]	Cross-sectional survey.	USA	1110 participants (671 women and 439 men)—data about women participants were included in the study.	Healthy reproductive-age women and men	Pubic hair preferences/symptoms	Pubic hair grooming and removal are prevalent among college-age individuals, with women more likely to engage in grooming and prefer a hair-free appearance. Genital itching is a common side effect associated with pubic hair grooming. The motivations for grooming vary, with women associating grooming with cleanliness, comfort, and sex appeal, while men may be influenced by partner preferences. Women’s total pubic hair removal was associated with younger age (*p* < 0.05); heterosexual orientation (*p* < 0.01); race/ethnicity (self-identified Asian/Asian American women and women in non-specified “other” race/ethnicity categories were significantly less likely to report complete hair removal as compared with white women; *p* < 0.01); and having either a monogamous (*p* < 0.01) or nonmonogamous sexual partner(s) (*p* < 0.01), as compared to having no sexual partner.	O
Crann et al. (2018) [26]	Cross-sectional online survey	Canada	1435	Healthy reproductive-age women	Vaginal health and hygiene practices	Widespread use of vaginal/genital hygiene products in Canada, with high self-reported rates of adverse symptoms (anti-itch creams, moisturisers/lubricants, gel sanitisers, feminine wipes, baby wipes, feminine washes/gels, and douches). Observed strong associations between product use and genital health complaints—though causal direction is uncertain (i.e., product use may follow symptoms rather than cause them).	O
DeMaria & Berenson (2013) [52]	Cross-sectional survey	USA	1677	Low-income women	Grooming among low-income women	Pubic hair grooming is pervasive among low-income women across racial backgrounds. Grooming behaviour varied significantly with race, age, income, body weight, and sexual history. The majority of women who had ever groomed indicated having used a razor and shaving cream for pubic hair grooming (77.2%; *n* = 1108), followed by trimming with scissors (23.1%; *n* = 392) and hair removal cream (18.7%; *n* = 268).	O
DeMaria et al. (2014) [49]	Cross-sectional survey	USA	333	Healthy reproductive-age women	Complications from pubic hair removal	Pubic hair removal is prevalent among women, with a significant proportion experiencing complications, particularly when using razors. Overweight/obese women and White women are more likely to report complications. There is a need for increased awareness and education on safe pubic hair removal practices.	O
DeMaria et al. (2016) [51]	Mixed-methods study combining quantitative and qualitative approaches	USA	663	Healthy reproductive-age women	Pubic hair grooming perceptions, mixed methods	Pubic hair removal is influenced by personal reasons and external factors, such as family, friends, and media. Initiation age, genital self-image, and sexual behaviours are significantly related to pubic hair removal. There is a need for further education regarding safe pubic hair removal methods, especially for those who initiate pubic hair removal and sexual behaviours concurrently. The most prevalent complications ever experienced due to pubic hair removal included razor burn (70.0%; *n* = 464) and ingrown hairs (69.8%; *n* = 463), with some participants reporting severe itching (31.1%; *n* = 206) and cuts (32.1%; *n* = 213). Those who identified as hair-free were significantly more likely to suffer from razor burn (*p* = 0.02), ingrown hairs (*p* = 0.02), and severe itching (*p* < 0.01).	O
Dendle et al. (2007) [54]	Case report	Australia	1	A 20-year-old woman	hair removing	Case underscores the need for caution and hygiene awareness—particularly for medically vulnerable individuals. Physicians should warn immunosuppressed patients of the risks of extensive body hair removal (in particular, removal of pubic hair) and suggest that they attend hygienic and reputable establishments. Advice on shaving techniques, ensuring that the wax is not too hot, and testing of products on nongenital areas can be offered. Patients should attend hygienic beauty salons, where therapists regularly wash their hands and wear gloves. Patient support groups, such as the Diabetes Council, should also be aware of and warn patients of the risks.	N
Foch et al. (2001) [38]	Cross-sectional, descriptive study	USA	169	Caucasian and African-American adolescent females (≤19 years)	Douching in adolescents	Sixty-nine % of participants reported vaginal douching, primarily for hygienic reasons (68%). Those who reported douching were more likely to have a history of sexual intercourse (*p* < 0.01) and a history of one or more sexually transmitted diseases (*p* < 0.05). The age at first douching correlated positively with the age at first sexual intercourse (r = 0.34, *p* < 0.001). African-Americans did not douche to a greater degree than Caucasians. However, racial differences were noted in knowledge of and attitude toward vaginal douching.	O
Güzel et al. (2011) [36]	Cross-sectional, observational study	Turkey	393	Reproductive-age women	Douching in rural Turkey	The major symptoms of the subjects were itching and vaginal discharge. Of the 393 women, 317 (80.66%) performed vaginal douching, and all of them had recurrent or treatment-resistant mixed-agent vulvovaginitis. The majority of the women douched for ritual cleansing or washing before prayer (n = 278; 91.6%). The majority of the cases (*n* = 354; 90.1%) were of lower socioeconomic and educational status. The odds ratios and 95% confidence interval (CI) of the risk variables—vaginal douching frequency, cervical motion tenderness, dyspareunia, and vaginal itching—were 9.39 (2.07–42.48), 7.31 (2.08–25.64), 6.52 (2.26–18.78), and 1.46 (1.22–1.74).	O
Heng L.S. et al. (2010) [32]	Cross-sectional, observational study	Cambodia	451	Reproductive-age women with normal and abnormal vaginal discharge and different douching habits	Douching and candidiasis	Vaginal douching is highly prevalent among Cambodian women—76.7% (*n* = 346). Douching was significantly more prevalent in urban than in rural women (85.7%, *n* = 198 vs. 67.3%, n = 148; *p* < 0.001). The practice is significantly associated with increased odds of vaginal candidiasis (dysuria with trichomoniasis (*p* = 0.061) and itching with BV (*p* = 0.063)).	O
Herbenick et al. (2010) [55]	Cross-sectional, internet-based survey	USA	2451	Sexually active women in US	US prevalence of pubic hair removal	Women reported a range of hair removal practices, from trimming to complete removal. Significantly associated with younger age, being partnered (not single or married), having received cunnilingus in past 4 weeks, recently examining one’s genitals, higher scores on genital self-image (FGSIS), and overall sexual function (FSFI; except orgasm subscale).	O
Herbenick et al. (2013) [56]	Prospective event-level daily diary study	USA	2453	Sexually active women in US	Pubic hair and sexual behaviour	A total of 15.2% of daily entries involved pubic hair removal, almost entirely shaving (~99%). Predictors of grooming: Events of grooming were significantly more likely on days when women reported younger age, interest in sex, vaginal fingering or finger-clitoral stimulation, casual partner contact, use of vaginal hygiene products, and application of creams to the genitals. Grooming was modestly linked to longer episodes of vaginal intercourse.	O
Holzman C. et al. (2001) [30]	Cross-sectional, multi-site epidemiologic study	USA	496	Symptomatic and asymptomatic women who douche	Risk factors associated with douching	Frequent douching correlated significantly with bacterial vaginosis even after adjusting for sexual risk factors. After adjustment for ethnicity and education, symptomatic bacterial vaginosis was more strongly associated with vaginal douching (OR = 5.8, 95% CI = 2.2, 16.6) than was asymptomatic bacterial vaginosis (OR = 2.6, 95% CI = 1.3, 5.2).	P
Klann et al. (2019) [24]	Cross-sectional study	USA (Boston University/Minnesota)	434	Women with vulvodynia	Vulvodynia and hygiene behaviours	Women with vulvodynia reported more frequent use of specialised vulvar cleansers and avoidance of soaps compared to controls. Wearing tight-fitting jeans or pants 4 times per week or more was crudely associated with vulvodynia.	O
Klebanoff et al. (2010) [33]	Prospective cohort study	USA	3620	Healthy reproductive-age women	Hygiene and BV	Vaginal douching is significantly associated with BV, suggesting a potential causal relationship. Other feminine hygiene behaviours assessed in this study were not strongly associated with BV, indicating that they may not be significant risk factors.	P
Leo & Benvenuti (2015) [27]	Combined in vitro laboratory assays and non-randomised clinical evaluation	Italy	2631	Reproductive age, pre-adolescents, adolescents, pre-menopausal and menopausal women	Plant-based cleansers	The experimental data showed the antibacterial, antimycotic, anti-inflammatory, and antioxidant activity of the natural principles contained in the four extracts. The clinical studies evidenced the significant reduction of genital signs and symptoms (itching, burning, erythema, oedema, vaginal dryness, dyspareunia) and the improvement of sexual female disorder. The cleanser selection should be tailored to the age and physiopathological condition of the woman.	O
McClelland et al. (2006) [35]	Prospective cohort study	Kenya	1270	Kenyan female sex workers enrolled between ~1992 and 2002, HIV-negative at baseline	Vaginal washing and HIV risk	Compared with women who did not perform vaginal washing, there was an increased risk for acquiring HIV-1 among women who used water (adjusted HR, 2.64; 95% CI, 1.00–6.97) or soap (adjusted HR, 3.84; 95% CI, 1.51–9.77) to clean inside the vagina. Women who performed vaginal washing with soap or other substances were at higher risk for HIV-1 compared with those who used water alone (adjusted HR, 1.47; 95% CI, 1.02–2.13).	P
McKee et al. (2009) [37]	Cross-sectional survey	USA	335	Reproductive-age women	Urban women’s hygiene practices	Prevalence of vaginal douching: Approximately 30.7% of women reported never having douched, while 51.1% of those who had ever douched indicated they no longer do. Ethnic Differences: Hispanic women were more likely to report never douching and to use imported products compared to Black women. Beliefs about douching: Women who currently douche held more positive beliefs about its benefits and safety—douching products are safe, douching promotes vaginal health, and that men find women who douche more attractive.	O
Mościcka et al. (2020) [44]	Cross-sectional questionnaire survey	Poland	140	Healthy reproductive-age women	Polish women’s hygiene during COVID	Many women reported a notable increase in showering or bathing immediately after returning home, as part of hygiene routines to reduce perceived infection risk. Slight decline in hair washing frequency, possibly due to reduced public appearances	O
Murina et al. (2014) [43]	Randomised controlled trial	Italy	32	Female patients diagnosed with vulvar dermatosis—either lichen sclerosus (LS) or lichen simplex chronicus (LSC)	Cleansing in vulvar dermatosis	No significant difference between cleansers in rubbing, applicability, or pleasantness ratings. Cleaner A (plant-based formula (almond, malva, jojoba oil, hyaluronic acid) BID + daily lanolin cream) users required significantly fewer lanolin applications (mean: 5.28 vs. 8.25; *p* < 0.001), indicating better symptomatic control compared to cleaner B (amino-acid surfactant with oat extract, maltodextrins, caprylic glycol, plus the same lanolin regimen). The aloe/jojoba/hyaluronic-based cleanser A might enhance skin soothing and hydration, reducing reliance on emollient rescue therapy.	O
Murina et al. (2020) [40]	Randomised, double-blind, controlled trial	Italy	40	Healthy reproductive-age women	Intimate hygiene and vulvar health	Both cleansers tested showed high performance for safety and tolerability on vulvar skin, but a plant-extract intimate cleanser (Saugella Hydraserum) may better support vulvar skin health compared to conventional lactic-acid washes—preserving hydration, maintaining optimal pH, and reducing sebum changes.	P
Ness R.B. et al. (2001) [28]	Cross-sectional analysis within a multicentre study	USA	654	Women with PID symptoms and douching habits	Douching and endometritis	Douching prior to/during PID treatment was associated with higher rates of endometritis and pelvic pain. Vaginal flora and douching (%): normal (28.2%), intermediate (36.3%), and bacterial vaginosis (50.8%) (*p* < 0.001).	P
Ness R.B. et al. (2002) [29]	Cross-sectional, multicentre study	USA	1200	Women with different douching habits and vaginal microfloral changes.	Douching and BV/lactobacilli	Douchers had an increased prevalence of bacterial vaginosis and reduced lactobacilli counts. Douching at least once per month was associated with an increased frequency of bacterial vaginosis. Those who douched recently (within 7 days) were at highest risk [odds ratio (OR) 2.1, 95% confidence interval (CI) 1.3, 3.1]. Douching for symptoms (OR 1.7, 95% CI 1.1, 2.6) and for hygiene (OR 1.3, 95% CI 1.0, 1.9), both related to bacterial vaginosis risk.	P
Rouzi et al. (2018) [48]	Cross-sectional survey	Saudi Arabia	400	Healthy reproductive-age women	Pubic hair removal complications in Saudi women	Pubic hair removal is a common practice among Saudi women, often initiated in early adolescence. While most complications are minor, a significant proportion of women experience issues requiring medical attention.	O
Ruiz et al. (2019) [25]	Descriptive observational	Brazil	220	Gynaecologists	Daily genital care in gynaecologists	Less than half adhered to ideal genital hygiene practices (use of water and soap), and nearly half reported vaginal discharge, indicating room for improvement.	O
Runeman et al. (2003) [46]	Experimental study with repeated measures	Sweden	12	Healthy reproductive-age women	Panty liners and vulvar microclimate	The use of conventional panty liners with non-breathable back sheets significantly alters the vulvar skin microclimate by increasing temperature and humidity and altering pH. Panty liners with breathable back sheets help maintain the vulvar skin microclimate closer to the natural state.	P
Runeman et al. (2005) [47]	Crossover experimental study with repeated measures	Sweden	32	Healthy reproductive-age women	Underwear tightness effect	Wearing tight-fitting string panties with liners had negligible effect on vulvar skin microclimate, pH, or aerobic microflora compared to standard underwear. It should be emphasised that the two pantyliners used in this study were both equipped with breathable back sheets. The assumption that tighter underwear would increase contamination from anorectal bacteria was not supported.	P
Schafer et al. (2002) [45]	Randomised controlled trial	Germany and Sweden	10	Healthy reproductive-age women	Skin barrier and occlusion	Vapour-permeable hygiene articles better maintain a stable skin barrier (lower overhydration and moisture retention) by reducing skin surface water loss and excessive stratum corneum hydration, which otherwise may render the skin more vulnerable to irritating agents or mechanical forces. Occlusion effects: Both pad type and environment significantly influence skin microclimate and moisture balance.	P
Shaaban et al. (2021) [34]	Cross-sectional observational study	Egypt	604	Reproductive-age women with IUCD	Douching in IUD users	Internal douching in IUCD users is strongly linked to higher rates of both historical (perform internal VD 260 (88.1%) vs. do not perform internal VD 151 (43.4%), *p* < 0.001) and current vaginal infections (perform internal VD 275 (91.05%) vs. do not perform internal VD 115 (38.1%), *p* < 0.001). BV is the most frequent infection, followed by Candida. The findings support public health advice discouraging intravaginal douching, especially for women with IUDs.	O
Sunay et al. (2011) [31]	Analytical cross-sectional observational study	Turkey	350	Reproductive-age women with normal and abnormal vaginal discharge and different douching habits	Douching and changes in vaginal discharge	Douching was significantly associated with demographic factors like marital status and income, and those who douched had nearly four times higher odds of abnormal vaginal discharge.	O
Trager (2006) [53]	Case report	USA	1	A 17-year-old woman	Review of hair removal issues: “pearls and pitfalls”	Hair removal may cause skin microtrauma and subsequent spread of infectious agents throughout the pubic area.	N
Yıldırım et al. (2020) [39]	Cross-sectional observational (descriptive).	Turkey	190	Women with different douching habits and vaginal microfloral changes.	Douching effect on vaginal microflora	While douching was not linked to current infection or measurable changes in flora, it was correlated with a greater likelihood of past genital infections. (*p* < 0.01).	O

P—positive; O—neutral; N—negative.

**Table 2:** Topics about washing and studies that included specific issues.

Topics	Articles Which Included Certain Topics, n = 21 (n/%)
**Douching**	15/71
- Reasons:	7/33
Personal hygiene	7/33
Healthy (get rid of infection, prevent infection)	7/33
To decrease vaginal symptoms (itching, burning)	4/19
- Association with vaginal microbiome changes and infection:	11/52
BV	7/33
Candida albicans	3/14
STI (*Neisseria gonorrhoea, Chlamydia trachomatis*)	2/10
Human immunodeficiency virus	1/5
**Bathing and showering**	5/24
Wash genitalia at least once a day.	4/19
I prefer shower over bathing as a routine washing method.	3/14
Increased bathing frequency is associated with BV development.	3/14
**Washing product usage**	7/33
Water influence on genitalia skin;	3/14
Specific intimate hygiene products (salvia, chamomile, non-irritating cleanser based on natural surfactants, lactic acid, lactose rum, etc.)	4/19

BV—Bacterial vaginosis; STI—sexually transmitted infections.

**Table 3:** Topics about underwear and studies that included specific issues.

Topics	Articles Which Included Certain Topics, *n* = 7 (*n*/%)
**Underwear**	3/43
Changing frequency	1/14
Association with BV and douching	2/29
Underwear material and healthy state	2/29
**Clothes and underwear tightness**	2/29
Association with BV	1/14
Association with vulvodynia	1/14
**Panty liners**	5/71
Changing frequency	1/14
Association with douching	1/14
Association with vaginal symptoms (pruritus, burning)	1/14
Influence of different types of panty liners on perineal skin	3/43

BV—Bacterial vaginosis.

**Table 4:** Topics about pubic hair removal and studies that included specific issues.

Topics	Articles Which Included Certain Topics, n = 11 (n/%)
**Pubic hair removing**	8/73
- Reasons:	
Aesthetics of the intimate area	5/46
Hygienic purposes	3/27
Self-confidence	2/18
- Influenced by	
Family	3/27
Friends	2/18
Social media	1/9
- Associated with	
Younger age	5/46
Sexual activity	3/27
**Preferred pubic hair removing methods**	9/82
Shaving	7/64
Waxing (hot/cold)	2/18
**Complications**	5/46
